# Evolution of tropical land temperature across the last glacial termination

**DOI:** 10.1038/s41467-022-32712-3

**Published:** 2022-09-02

**Authors:** M. H. Løland, Y. Krüger, A. Fernandez, F. Buckingham, S. A. Carolin, H. Sodemann, J. F. Adkins, K. M. Cobb, A. N. Meckler

**Affiliations:** 1 Department of Earth Sciences, University of Bergen, Bergen, 5007 Norway; 2 Bjerknes Centre for Climate Research, Bergen, 5007 Norway; 3 Andalusian Institute of Earth Sciences, CSIC-University of Granada, Granada, Spain; 4 Department of Earth Sciences, University of Oxford, Oxford, UK; 5 Department of Earth Sciences, University of Cambridge, Cambridge, UK; 6 Geophysical Institute, University of Bergen, Bergen, 5007 Norway; 7 Division of Geological and Planetary Sciences, California Institute of Technology, Pasadena, CA USA; 8 Department of Earth and Atmospheric Sciences, Georgia Institute of Technology, Atlanta, GA USA

**Keywords:** Palaeoclimate, Climate sciences, Geology

## Abstract

The tropical West Pacific hosts the warmest part of the surface ocean and has a considerable impact on the global climate system. Reconstructions of past temperature in this region can elucidate climate connections between the tropics and poles and the sensitivity of tropical temperature to greenhouse forcing. However, existing data are equivocal and reliable information from terrestrial archives is particularly sparse. Here we constrain the magnitude and timing of land temperature change in the tropical West Pacific across the last deglaciation using an exceptionally precise paleothermometer applied to a well-dated stalagmite from Northern Borneo. We show that the cave temperature increased by 4.4 ± 0.3 °C (2 SEM) from the Last Glacial Maximum to the Holocene, amounting to 3.6 ± 0.3 °C (2 SEM) when correcting for sea-level induced cave altitude change. The warming closely follows atmospheric CO_2_ and Southern Hemisphere warming. This contrasts with hydroclimate, as reflected by drip water δ^18^O, which responds to Northern Hemisphere cooling events in the form of prominent drying, while temperature was rising. Our results thus show a close response of tropical temperature to greenhouse forcing, independent of shifts in the tropical circulation patterns.

## Abstract (web summary)

Ancient drip water in a Borneo stalagmite reveals a strong land temperature rise across the last glacial termination in close correspondence with atmospheric CO2, and an intriguing decoupling between tropical temperature and hydroclimate.

## Introduction

The evolution of climate from the Last Glacial Maximum (LGM) to the Holocene brought major changes in Earth’s climate, including a large increase in atmospheric CO_2_ concentration from ∼200 to ∼280 ppmv^1,2^. Understanding deglacial climate changes in different regions of the world can improve our understanding of the complex connections between different components of the climate system, and the sensitivity of climate to greenhouse forcing. Much attention has therefore been paid to reconstructing and understanding the deglacial evolution of CO_2_, ice sheets, and ocean circulation. However, to further improve our understanding of past climate variations, including regional implications, we are lacking precise and accurate temperature records from land and low latitudes.

The tropics have a considerable impact on the global climate system, but the magnitude of this region’s glacial-interglacial temperature change, and its response across millennial-scale events of the last deglaciation, remain equivocal. Existing proxy data show a large range of deglacial warming estimates in low latitudes (e.g., compiled by ref. 3) from 1–6 °C, whereas climate model estimates range from 1.6 to 3.5 °C deglacial warming^4^. The evolution of sea surface temperature (SST) changes across the last deglaciation also differs considerably among different locations even in the same region, such as the tropical West Pacific^5^. Therefore, it remains uncertain whether temperature in this region responds to Northern Hemisphere (NH) abrupt climate events, changes in the Southern Hemisphere (SH), greenhouse gases, or a combination of these. This ambiguity is reinforced by the dominance of marine paleoclimate records as there are few robust methods to reconstruct temperature on land, a major obstacle for our understanding of the role of the tropics in deglacial climate evolution.

Fluid inclusion (FI) microthermometry has been proposed as a quantitative thermometer to reconstruct paleotemperatures from stalagmites^6^. The method is based on the physico-chemical properties of drip water relics trapped in microscopic fluid inclusions that form during stalagmite growth. If the physical and chemical properties of the enclosed drip water are preserved, the density of the water is directly related to the cave temperature at the time when the inclusion sealed off from the environment. The density of the inclusion water can be deduced from measurements of its liquid-vapour homogenisation temperature by using a thermodynamic model that accounts for surface tension effects^7^. The strength of stalagmite fluid inclusion microthermometry lies in the purely physical basis of the method. The approach does not require any empirical calibration, which is needed for most other proxies and can be associated with many uncertainties.

Here, we derive a quantitative tropical cave temperature record across the last deglaciation based on stalagmite SC02 from Northern Borneo. Each data point in our record represents the mean value of ∼30–40 individual fluid inclusion analyses from the same growth layer, showing near Gaussian distributions (Supplementary Fig. 1) with standard deviations (SD) of 0.5 to 1.8 °C (average: 1.0 °C). The resulting uncertainties of the averaged cave temperatures, reported as two standard errors of the mean (2 SEM), range from 0.2 °C to 0.4 °C. Due to year-round stable cave temperatures (Methods), our record provides a rare estimate of low-latitude terrestrial temperature not affected by seasonality. The cave temperatures reflect average outside air temperatures, with minor offsets likely caused by evaporative cooling, assumed to be stable over time (Methods).

## Results and discussion

### Deglacial evolution of tropical temperature

**Fig. 1:**
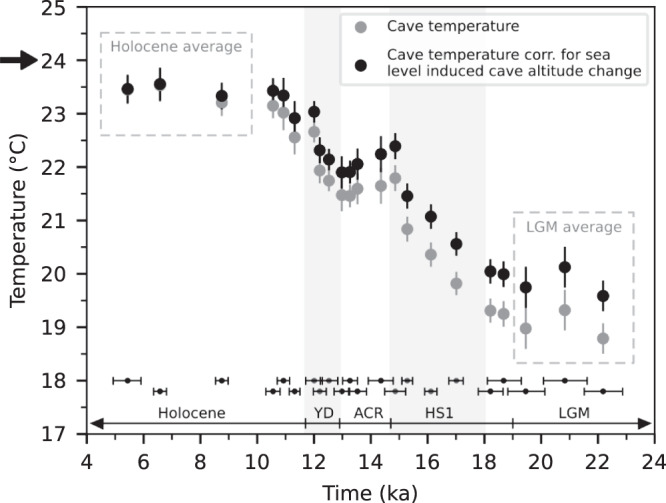
Reconstructed cave temperatures from stalagmite SC02 by means of fluid inclusion microthermometry. Grey circles are measured cave temperatures derived from ~30 – 40 fluid inclusion analyses at each sample position, while black circles represent the corresponding sea level corrected temperatures. Each temperature point integrates over a time period between 50 and 250 years, depending on the width of the analysed layers and the stalagmite growth rate. Error bars are two standard errors of the mean (2 SEM). U/Th ages (with 95% confidence intervals) from ref. 28 are shown below. The magnitude of glacial-interglacial temperature change (ΔT) is 4.4 ± 0.3 °C (2 SEM) in the cave, and 3.6 ± 0.3 °C (2 SEM) when corrected for sea level induced changes in cave altitude. ΔT is calculated as the difference between averaged LGM (>19 ka) and Holocene (<10 ka; after main sea level rise^8^) temperatures, indicated by dashed boxes. Present-day cave temperature is 24.0 °C (black arrow). Grey vertical bars indicate pronounced NH cooling episodes. LGM Last Glacial Maximum, HS1 Heinrich stadial 1, ACR Antarctic Cold Reversal, YD Younger Dryas.

We find a clear glacial-interglacial temperature contrast suggesting deglacial warming (ΔT) in the cave of 4.4 ± 0.3 °C (2 SEM) from 19.0 ± 0.2 °C (2 SEM) at the LGM to 23.4 ± 0.2 °C (2 SEM) in the Holocene (Fig. 1). To derive the climatic component of the deglacial warming, the measured cave temperatures were corrected for the secondary cooling effect induced by glacial sea level lowering. To this end, we used the sea level reconstruction of ref. 8 and a lapse rate of surface air temperature of 0.6 °C/100 m observed in the area today (Methods). The resulting altitude-corrected deglacial temperature change reduces to 3.6 ± 0.3 °C (2 SEM).

**Fig. 2:**
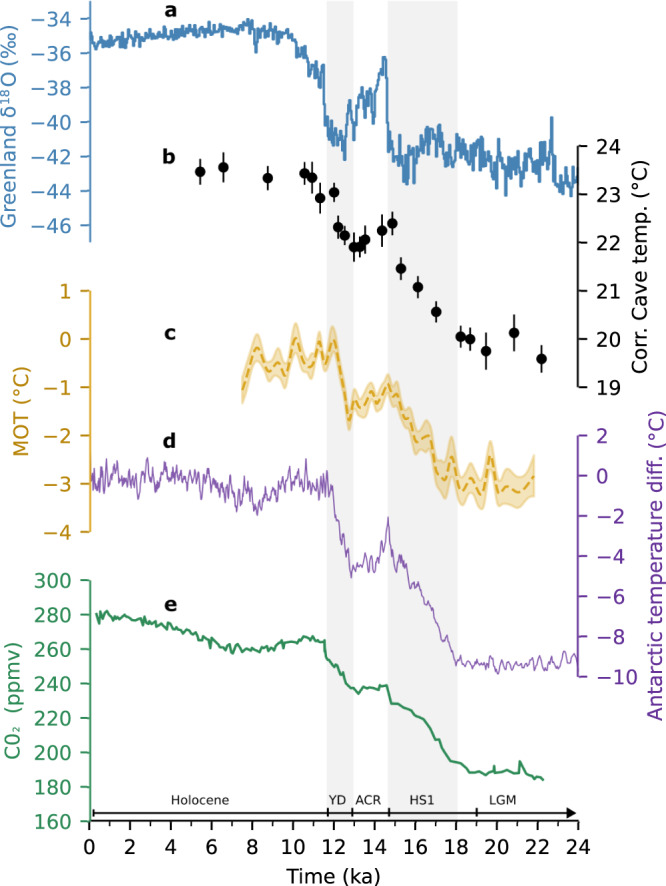
Comparison of Borneo land temperature with other paleoclimatic records of the last deglaciation. **a** Greenland ice core (NGRIP) δ^18^O reflecting Greenland temperature^44^. **b** Cave temperature record from this study corrected for changes of cave altitude relative to sea level, with 2 SE error bars. **c** Mean global ocean temperature (MOT) with 1σ-uncertainty band, derived from noble gas concentrations of air inclusions in ice cores^10^ plotted on the WD 2014 age scale. **d** Antarctic temperature difference from the average of the last 1000 years^9^ with a 5-point moving average. **e** Atmospheric CO_2_ concentration^1,2^. **d**, **e** are plotted on the AICC2012 age scale^45^. Grey vertical bars indicate pronounced NH cooling episodes. LGM Last Glacial Maximum, HS1 Heinrich stadial 1, ACR Antarctic Cold Reversal, YD Younger Dryas.

We find remarkable similarity both in the structure and timing between our cave temperature record and Antarctic ice core records of SH temperature^9^ and atmospheric CO_2_ concentration^1,2^ (Fig. 2d, e). This includes the early increase of temperature and CO_2_ concentrations during Heinrich stadial 1 (HS1), a slight decrease during the Antarctic Cold Reversal (ACR), and the second increase during the Younger Dryas (YD). Even an apparent acceleration of warming at the end of HS1 and the YD in our Borneo record is consistent with sudden rises of atmospheric CO_2_ (Fig. 2e). We thus find clear evidence that land temperature on Borneo did not decrease during North Atlantic abrupt cooling events such as HS1 and YD. Similar to land temperature on Borneo, mean global ocean temperature (MOT)^10^ is closely correlated with SH warming and atmospheric CO_2_ (Fig. 2c), suggesting that these records represent the globally dominant timing of deglacial temperature rise. Interestingly, reconstructions of mean global surface temperature based on compilations of proxy records and/or paleo data assimilation into climate models^11,12^ suggest a slightly different timing of global deglacial warming (Supplementary Fig. 2). This discrepancy raises the question of whether there could be an overrepresentation of the NH influence in these records^11,12^.

Our estimate of the magnitude of deglacial warming of about 3.6 °C is in line with a recent proxy- and model^12^ based estimate for tropical warming of 3.4 °C (15° S to 15°N, calculated for the same time slices) and land temperature reconstructions based on tropical African lakes^13,14^. However, this deglacial warming is significantly larger than the ∼1– 2 °C estimated by earlier SST compilations^15,16^ and substantially lower than ∼6 °C low latitude land temperature warming inferred from noble gas concentrations in groundwater^17^. Regionally in the tropical West Pacific, our estimate is consistent with a clumped isotope-based estimate for ΔSST of ∼4 °C^3^. Mg/Ca derived SST records from the region suggest slightly less deglacial warming of 2–3 °C^5,18–20^, but these estimates contain additional uncertainty due to potential biases by deglacial changes in ocean pH and/or salinity^21,22^. In absolute terms, our cave temperatures are systematically lower than reconstructed SSTs from the region (Supplementary Fig. 3). Similar differences are also observed today and are likely due to larger heat capacity and lower albedo of the ocean compared to land

While Mg/Ca-based SST records from different parts of the tropical West Pacific are similar in the suggested amplitude of deglacial warming, they diverge regarding the structure of deglacial warming (ref. 5; Supplementary Fig. 3), which has prevented deriving a clear picture of the temporal evolution of warming in this region. Records close to the open tropical West Pacific and the Eastern Indian Ocean^5,23^ show a step-like evolution with SH timing, characterized by increasing temperatures during HS1, a slight decrease during the ACR, and a subsequent increase during the YD, similar to our record. In contrast, locations in the Indonesian Throughflow region, closer to Borneo, suggest a steady increase in temperature over the same time period^5,19^. The agreement between our land temperature record from Borneo and the “open ocean” records indicates that the step-like, SH-paced temperature evolution is representative for the whole region, and suggests that the marine SST records from within the archipelago might be impacted by additional influences such as seasonality^5^ or sea level-induced changes in circulation affecting SST and/or salinity.

### Decoupling of temperature and hydroclimate

**Fig. 3:**
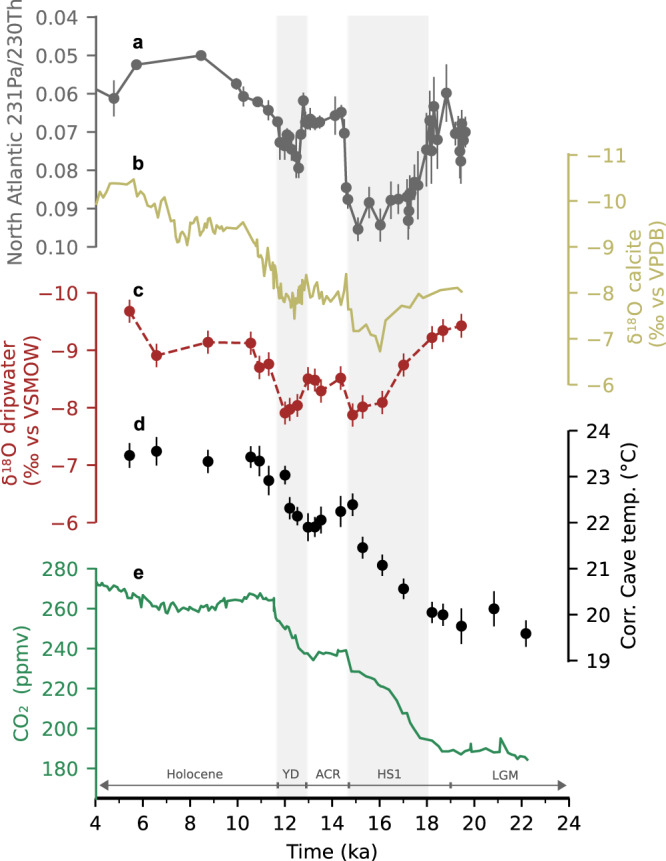
Deglacial evolution of Borneo temperature and hydroclimate in comparison to North Atlantic and greenhouse forcing. **a** North Atlantic overturning circulation changes based on ^231^Pa/^230^Th from sediment core OCE326-GGC5^24^ with 2 SD error bars. **b** δ^18^O_cc_ of stalagmite SC02 (10–20 ka: ref. 28; Holocene: this study). **c** Calculated δ^18^O_dw_ based on δ^18^O_cc_ and cave temperature, corrected for ice volume related changes of mean ocean δ^18^O, with 2 SE error bars (Methods and Supplementary Data). **d** Cave temperature record from this study corrected for changes of cave altitude relative to sea level, with 2 SE error bars. **e** Atmospheric CO_2_ concentration^1,2^. Grey vertical bars indicate pronounced NH cooling episodes. LGM Last Glacial Maximum, HS1 Heinrich stadial 1, ACR Antarctic Cold Reversal, YD Younger Dryas.

During the last deglaciation, NH climate was characterized by abrupt cooling and warming episodes, while the SH showed an offset behaviour with steady warming during NH cold events and stable temperature during NH warming (Fig. 2a, e). The NH cooling events were associated with reduced Atlantic meridional overturning circulation^24^ with repercussions also in low latitude climate (e.g., ref. 25), and are clearly reflected in tropical stalagmite oxygen isotope (δ^18^O_cc_) records (e.g., refs. 26,27), interpreted as records of hydroclimate variability. This is also true for the δ^18^O_cc_ record of stalagmite SC02^28^ (Fig. 3b). In contrast, our SC02 temperature record does not indicate any cooling response to NH cooling and instead closely follows CO_2_ and SH warming (Figs. 2 and 3). This discrepancy between δ^18^O_cc_ and temperature is at first sight surprising, given that δ^18^O_cc_ is also influenced by temperature, in addition to the hydroclimate-related variations in the oxygen isotope signal of the drip water (δ^18^O_dw_).

With temperature and δ^18^O_cc_ from the same stalagmite we have calculated δ^18^O_dw_ (Methods), providing a more direct constraint on the hydroclimate signal than δ^18^O_cc_ (Fig. 3). On Borneo today, δ^18^O_dw_ closely follows rainwater δ^18^O and regional precipitation amounts^29,30^. When corrected for ice sheet-related global changes in seawater δ^18^O (∼1.0‰ at the LGM^31^) we obtain similar δ^18^O_dw_ values for the LGM (9.43‰, >19ka; *N* = 1) and the Holocene (9.24‰, averaged <10ka; *N* = 3), supporting earlier studies that suggested only minor changes in regional hydroclimate on Northern Borneo when comparing LGM and the Holocene^32^. This is different from other parts of the Indo-Pacific Warm Pool, where both reconstructions and model simulations suggest a spatially variable LGM-vs-Holocene response^33^. The lack of clear LGM-vs-Holocene change in Northern Borneo δ^18^O_dw_ stands in stark contrast to the strong δ^18^O_dw_ enrichment of up to −7.87 ‰ during the glacial termination, associated with NH cooling events during HS1 and YD. Assuming precipitation amount as the major influence on δ^18^O_dw_, this deglacial evolution of δ^18^O_dw_ corroborates previous interpretations of transient drying in the northern Sunda Shelf area based on speleothem δ^18^O_cc_ records^26,28^. This finding is consistent with global-scale shifts in tropical rainfall during episodes of hemispherically asymmetric temperature changes associated with latitudinal shifts in atmospheric circulation patterns such as the Intertropical Convergence Zone^23,25,34^.

Overall, our results add robust constraints on the sensitivity of tropical climate to greenhouse forcing and high-latitude climate. Land temperature on Borneo responded with 3.6 ± 0.3 °C warming to the 80ppm LGM – Holocene change in atmospheric CO_2_. With hydroclimate and temperature information from the same archive, we see a clear decoupling of these climate parameters. The primary control on Borneo temperature appears to be greenhouse forcing with a SH timing. In contrast, regional hydroclimate, as reflected by δ^18^O_dw_, did not detectably respond to greenhouse forcing but instead reacted strongly to the hemispherically asymmetric millennial-scale NH cooling events and associated changes in atmospheric circulation patterns. The temperature record from Borneo likely reflects the enormous heat reservoir of the surrounding Indo-Pacific Warm Pool, a major source for heat and moisture to the extra-tropics. Our data thus show a different deglacial evolution of the availability of this heat and moisture on the one hand and its fate on the other hand, with the former being dictated by the south and the latter by inter-hemispheric temperature gradients responding to changes in the north.

## Methods

### Present-day climate at the study site

The stalagmite sample SC02 was collected in 2006 from Secret Chamber in the Clearwater Cave system of Gunung Mulu National Park, Northern Borneo (4°N, 115°E). Borneo is located in the tropical West Pacific, an area that hosts the warmest temperature in the world’s oceans (Supplementary Fig. 3e). As the Intertropical Convergence Zone (ITCZ) annually migrates north and south, the placement of the cave just north of the equator allows it to remain within the West Pacific deep tropical convection year-round. Gunung Mulu receives an annual rainfall average of 5000 mm with weak seasonality due to its position within the ITCZ; however, the site is significantly impacted by El Niño Southern Oscillation (ENSO) dynamics, which exerts a dominant control on interannual precipitation variability^29^.

The present-day cave temperature in Secret Chamber is 24.0 °C (ranging from 23.9 °C to 24.1 °C), based on temperature time series from data loggers (Van Essen CTD-Divers) placed at two locations in the cave between April 2018 to April 2019. The cave temperature in Secret Chamber is close to the inner cave temperature in nearby Lang’s Cave (28 m a.s.l.), a tourist cave in Gunung Mulu National Park where both inner cave and cave entrance temperatures have been measured in multiple years using Onset HOBO U23-001 ProV2 loggers (Supplementary Table 1). In 2018, the loggers were cross-calibrated before deployment. In Lang’s Cave, inner cave temperatures were consistently slightly lower (by 0.4 °C) than those measured at the cave entrance. The temperature at the entrance of Lang’s Cave, in turn, is similar to the average temperature measured at nearby Mulu airport (Supplementary Table 1). The slightly colder temperatures in the inner caves might be due to evaporative cooling in the cave system. We note that regional SST is substantially higher (28 – 29 °C) than inland temperatures on Borneo^35^ due to the larger heat capacity and lower albedo of the ocean compared to land. The warm surface ocean affects meteorological station data close to the coast^35^ but does not reach as far inland as our study site. In addition, the rainforest likely has a cooling effect through evapotranspiration and radiative shielding. Available evidence^36^ suggests that in Northern Borneo, vegetation did not change much since the LGM, suggesting that any temperature effect of the vegetation remained similar to today.

### Sample and sample preparation

SC02 is a ∼580 mm long stalagmite covering the time period from the last interglacial to the late Holocene. In the present study, a 204 mm long part of the stalagmite was investigated that grew between ∼5.4 to 23 ka. This part of SC02 exhibits an average growth rate of 11 µm/year and a columnar calcite fabric^37^. The columnar crystal units display uniform crystallographic orientation and build up large composite crystals of up to one centimetre width. The fluid inclusions are inter-crystalline (open columnar fabric) and formed by incomplete lateral coalescence of adjacent columnar crystals^38^. The inclusions are characteristically monophase liquid and elongate parallel to the stalagmite growth direction. Occasionally two-phase liquid-vapour inclusions occur but these were not considered for microthermometric measurements because their bulk water densities are not related to the formation temperature. The size of the inclusions analysed in this study ranges from 10^2^ – 10^5^ µm^3^.

A 13 mm thick slab of SC02 that previously had been used for dating and stable isotope analyses (Supplementary Fig. 4a) was used as sample material in the present study. For microthermometric analyses we cut out blocks from the side of this slab, fixed them on glass slides with super glue, and prepared ∼300 µm thick sections using a low-speed rock saw (Buehler Isomet). A diamond wire saw (Well 3241) was used to divide the sections in 5 mm wide stripes (Supplementary Fig. 4b) before removing them from the glass substrate in acetone. Fluid inclusion measurements were performed on fragments split from the 5 mm wide stripes of the unpolished sections, using immersion oil to make them transparent for microscopic observation in transmitted light.

Since the microthermometric measurements were performed on the side of the stalagmite slab the layers were traced to the centre to determine the exact age and the corresponding δ^18^O_cc_ for each sampling position.

### Fluid inclusion microthermometry

In the present study we used classical fluid inclusion microthermometry to determine stalagmite formation temperatures^6^. The underlying temperature proxy is the density of former drip water that is preserved in microscopic cavities in the stalagmite. These fluid inclusions form during the calcite growth and the density of the enclosed water is assumed to relate directly to the cave temperature at the time the inclusions sealed off from the environment. The microthermometry approach is based on temperature measurements of the liquid-vapour homogenisation, i.e., the transition from a two-phase liquid-vapour to a monophase liquid state, which is a measure of the water density. Since fluid inclusions in stalagmites are characteristically monophase liquid, and spontaneous nucleation of the vapour phase fails due to long-lived metastability^39,40^, we used single ultra-short laser pulses to stimulate vapour bubble nucleation in the metastable state of the liquid water^41^. Once the vapour bubble has formed, the water in the inclusion is in a stable liquid-vapour equilibrium state. Upon subsequent heating, the liquid phase expands at the expense of the vapour bubble. Eventually, the bubble collapses and the Inclusions homogenise to the liquid phase (L + V → L) at T_h(obs)_ (Supplementary Fig. 5). The measured T_h(obs)_ values were corrected for the volume-dependent effect of surface tension on liquid vapour homogenisation^7^ by calculating T_h∞_, the homogenisation temperature of a hypothetical, infinitely large inclusion. If the inclusions have preserved their original water density, T_h∞_ is equal to the formation temperature T_f_ of the stalagmite when the inclusion closed off. The calculation of T_h∞_ requires an additional measurement of the vapour bubble radius based on bubble images taken at a known temperature.

### Experimental setup

Microthermometric measurements were performed with a Linkam THMSG600 heating/cooling stage mounted on an Olympus BX51 microscope. The microscope was equipped with a LMPLFLN 100x/0.8 objective and a Leica DFC350 FX digital camera for visual observation of the inclusions in transmitted light. Synthetic H_2_O and H_2_O–CO_2_ fluid inclusion standards were used for temperature calibration, yielding an accuracy of the heating/cooling stage of ±0.1 °C. An amplified TI:Sapphire laser (CPA-2101, Clark-MXR, Inc) coupled into the microscope provided the ultra-short laser pulses for stimulating vapour bubble nucleation in the metastable liquid inclusions. The principle of the analytical setup is illustrated in ref. 41.

### Microthermometry measurements

At each sample position ~30 – 40 fluid inclusions were analysed, including duplicate measurements of T_h(obs)_ for each inclusion with a reproducibility of typically ±0.05 °C, and 3–4 bubble radius measurements derived from different images taken at the same temperature. A cut-off value of 27 °C was used for the microthermometric measurements to avoid irreversible volume alterations of other inclusions in the sample caused by fluid overpressure that builds up with increasing overheating of the inclusions above formation temperature. Inclusions that homogenise above 27 °C are most likely affected by volume alterations resulting from sample preparation, and thus were ignored for cave temperature reconstruction.

The T_h∞_ values obtained at the individual sample positions display Gaussian-like distributions with one standard deviations (1 SD) ranging from 0.54 to 1.83 °C. The average standard deviation of 1.0 °C was used as basis for a 3σ criterion to classify outliers. On average 3% of the inclusions were classified as outliers (deviating more than ±3 °C from the mean), and on average 6% when including the ones that did not homogenise below 27 °C (Supplementary Fig. 1). The mean value of the T_h∞_ distribution after outlier removal is considered as the best estimate of the actual stalagmite formation temperature, i.e., cave temperature. Two standard errors of the mean (2 SEM) for each layer range between 0.2 and 0.4 °C.

The effect of sea level induced changes of the cave altitude on the cave temperature was calculated using the sea level reconstruction of ref. 8 and a lapse rate of surface air temperature of 0.6 °C/100 m (see below). A Monte Carlo approach (*N* = 100,000) was used to propagate errors of the cave temperatures and the sea level reconstruction, and combined errors are reported as 2 SEM. The magnitude of glacial-interglacial temperature change (ΔT) was calculated as the difference between the average LGM (>19ka; *N* = 3) and Holocene (<10 ka, *N* = 3) temperatures. The reported uncertainty (2 SEM) of ΔT was estimated using a Monte Carlo simulation (*N* = 100,000) based on the observed distribution statistics of the respective data points.

### Lapse rate for altitude change correction

The atmospheric temperature change with changing sea level was estimated from present-day climatological information from ERA5 reanalysis data^42^ in the vicinity of the cave location. To this end, 3-hourly mean surface layer (2 m above ground) temperatures were extracted for the period 1980–2019 at each grid point in an area around Mulu (114–118°E, 2.0–4.5°N), averaged to monthly values, and plotted at the respective elevation (ranging between 0 and 1278 m.a.s.l). The 2 m air temperatures reflect the effect of sensible and latent heat fluxes from the underlying surface, that are also expected to affect the cave temperature. Elevation was calculated using the hypsometric equation with a scale height of 8000 m and the surface pressure from ERA5. A linear fit to the binned surface-layer temperatures (bin interval 100 m) provides a lapse rate of Γ = 0.61 K/100 m, with a 95% confidence interval of ±0.002 K/100 m (Supplementary Fig. 6). The lapse rate obtained this way is very similar to the slope of the temperature profile in the free atmosphere at a location with low topography (114.15°E, 4.00°N, 32 m.a.s.l.) for the same period and dataset (Supplementary Fig. 6, black line).

We apply the present-day lapse rate when correcting for changes in cave altitude, assuming that the lapse rate remained roughly similar to today. This assumption is supported by the similarity of δ^18^O_dw_ between LGM and Holocene, as well as evidence for rainforest vegetation in Northern Borneo at the LGM^36^. An exception could be HS1 and YD, where climate was probably drier than today. A hypothetical (large) increase in the lapse rate by 0.2 K/100 m would lead to a stronger altitude correction, increasing our corrected temperatures by up to 0.2 °C and 0.1 °C during HS1 and YD, respectively.

### Stable isotope measurements

The stable isotope measurements in SC02 between 19.5–10.7ka were performed in the study of ref. 28. Samples were milled along the growth axis of SC02 in the Department of Earth Sciences at the University of Oxford. Samples were milled in 1 mm increments and increased to 0.2 mm intervals around the Younger Dryas section. Analyses were performed on a Delta V Advantage isotope ratio mass spectrometer coupled to either a Kiel IV carbonate device or Gas Bench II introductory system at the University of Oxford. Holocene δ^18^O_cc_ measurements were added as part of this study (Supplementary Fig. 4). Samples were taken from the top of SC02 to 10.7 ka (99 samples along the growth axis of SC02) in increments of 1–2 mm. Analyses were performed using a Thermo Fisher Scientific MAT-253 isotope ratio mass spectrometer coupled to a Thermo Fisher Scientific Kiel IV carbonate preparation device in the Facility for Advanced Isotopic Research (FARLAB) at the University of Bergen.

### Calculation of drip water δ^18^O

The calculation of δ^18^O_dw_ is based on δ^18^O_cc_ and the corresponding (uncorrected) cave temperatures using the inverted temperature calibration of ref. 43 (1). In this equation, δ^18^O of calcite and water are given relative to VSMOW and *T* is the temperature in Kelvin.

1 $${{{{{{\rm{\delta }}}}}}}^{18}{O}_{{dw}}=\,{e}^{\left(\frac{1000\times {{{{{\rm{ln}}}}}}\left(1000+{{{{{{\rm{\delta }}}}}}}^{18}{O}_{{cc}}\right)+24,60-\frac{16,10\times 1000}{T+273,15}}{1000}\right)}-1000$$

In addition, the resulting δ^18^O_dw_ values were corrected for a 1.0 ‰ change in mean ocean δ^18^O from the LGM to the Holocene^31^ due to continental ice decay. This correction makes use of the same sea level curve of ref. 8 as the correction of cave temperatures. A Monte Carlo approach (*N *= 100,000) was used to propagate errors of the cave temperatures, the δ^18^O_cc,_ and the sea level reconstructions. The combined error of δ^18^O_dw_ is dominated by the uncertainty in δ^18^O_cc_. Although the analytical error of δ^18^O_cc_ was better than 0.1‰ (1 SD), we used a conservative error of 0.2‰ (2 SD) to take into account some uncertainty in the exact isotopic composition of the area investigated with microthermometry.

## Supplementary information

Supplementary Information

Supplementary Data

## Data Availability

The data generated in this study have been deposited in the PANGEA database under accession code [to be added].
