# Supplementary material for: Evolution of tropical land temperature across the last glacial termination
Source: Nat Commun. 2022 Sep 2;13:5158. doi: 10.1038/s41467-022-32712-3 (PMC9440061; doi:10.1038/s41467-022-32712-3)
Supplement: Supplementary file 1 — Supplementary Information [file 41467_2022_32712_MOESM1_ESM.pdf]

Supplementary Information

Evolution of tropical land temperature across the last glacial termination

M. H. Løland[1,2*], Y. Krüger[1], A. Fernandez[3], F. Buckingham[4], S. A. Carolin[5], H. Sodemann[2,6], J. F. Adkins[7], K. M. Cobb[8], A. N. Meckler[1,2]

[1]Department of Earth Sciences, University of Bergen, Bergen, 5007, Norway

[2]Bjerknes Centre for Climate Research, Bergen, 5007, Norway

[3]Andalusian Institute of Earth Sciences, CSIC-University of Granada, Granada, Spain

[4]Department of Earth Sciences, University of Oxford, Oxford, UK

[5]Department of Earth Sciences, University of Cambridge, Cambridge, UK

[6]Geophysical Institute, University of Bergen, 5007, Norway

[7]Division of Geological and Planetary Sciences, California Institute of Technology, Pasadena, USA

[8]Department of Earth and Atmospheric Sciences, Georgia Institute of Technology, Atlanta, USA

*Corresponding author: Marit H. Løland (marit.loland@uib.no)

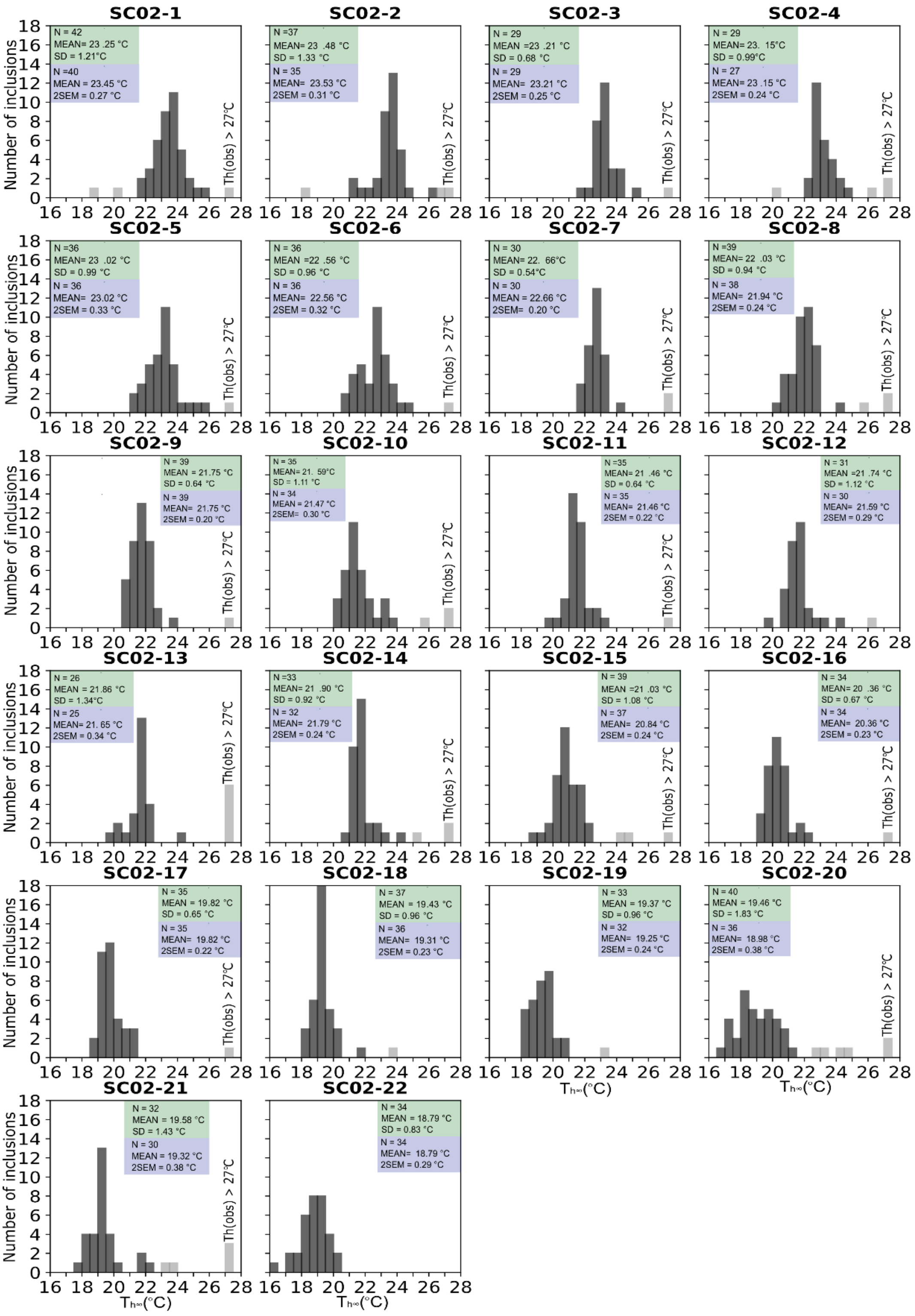

Supplementary Figure 1. Distributions of calculated $T_{h\infty}$ values derived from microthermometric measurements for 22 sample positions of stalagmite SC02. Sample ID is indicated above each histogram. Green boxes show the total sample size (N), the mean value, and the standard deviation (1 SD) of the $T_{h\infty}$ distribution. Note, inclusions with $T_{h(obs)} > 27$ °C were not analysed to avoid high fluid overpressure in other inclusions in the same sample. They were not included in the statistical analyses. Blue boxes show the sample size (N), mean value, and 2 standard errors of the mean (2 SEM) as used in the main figures, calculated from the distributions excluding outliers that deviate more than ± 3.0 °C from the mean. These outliers and the inclusions with $T_{h(obs)} > 27$ °C are marked in grey.

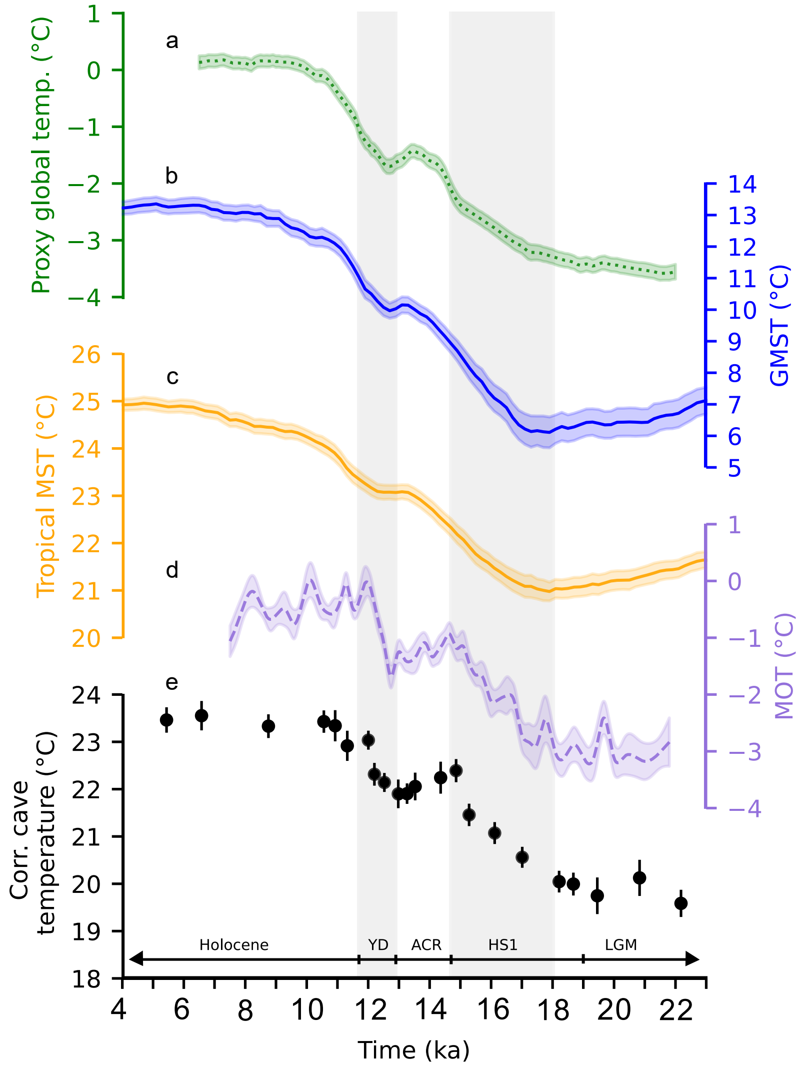

Supplementary Figure 2. Comparison of Borneo temperature with global and tropical temperature estimates. a) Global proxy temperature stack shown as deviations from the early Holocene (11.5 – 6.5 ka) mean, with error (1 SD)[1]. b) Global mean surface temperature (GMST) with error (1 SD) from a climate model reanalysis of global proxy SST[2]. c) Tropical (15° S to 15° N) mean surface temperature (MST) with error (1 SD) calculated from the gridded output of ref.[2]. d) Mean global ocean temperature (MOT) with 1σ-uncertainty band, derived from noble gas concentrations of air inclusions in ice cores[3] plotted on the WD 2014 age scale. e) Cave temperature record from this study corrected for changes of cave altitude relative to sea level. Error bars are 2 SEM. Grey vertical bars indicate pronounced NH cooling episodes. LGM: Last Glacial Maximum, HS1: Heinrich stadial 1, ACR: Antarctic Cold Reversal, YD: Younger Dryas. Differences in timing become apparent during the period between 15 and 11 ka: The global surface temperature records (a, b) show rising temperatures during the ACR whereas Borneo temperature and MOT decline, following Antarctic temperature and CO_2 (Fig. 2). This difference suggests that the global temperature records are influenced by a combination of NH and SH signals, whereas Borneo temperature and MOT strictly follow the SH timing. The discrepancy between the reanalysis dataset (b) and our Borneo record does not appear to be solely due to the larger region represented by the former, as the same discrepancy is apparent when extracting tropical data only from the reanalysis product of ref.[2] (panel c).

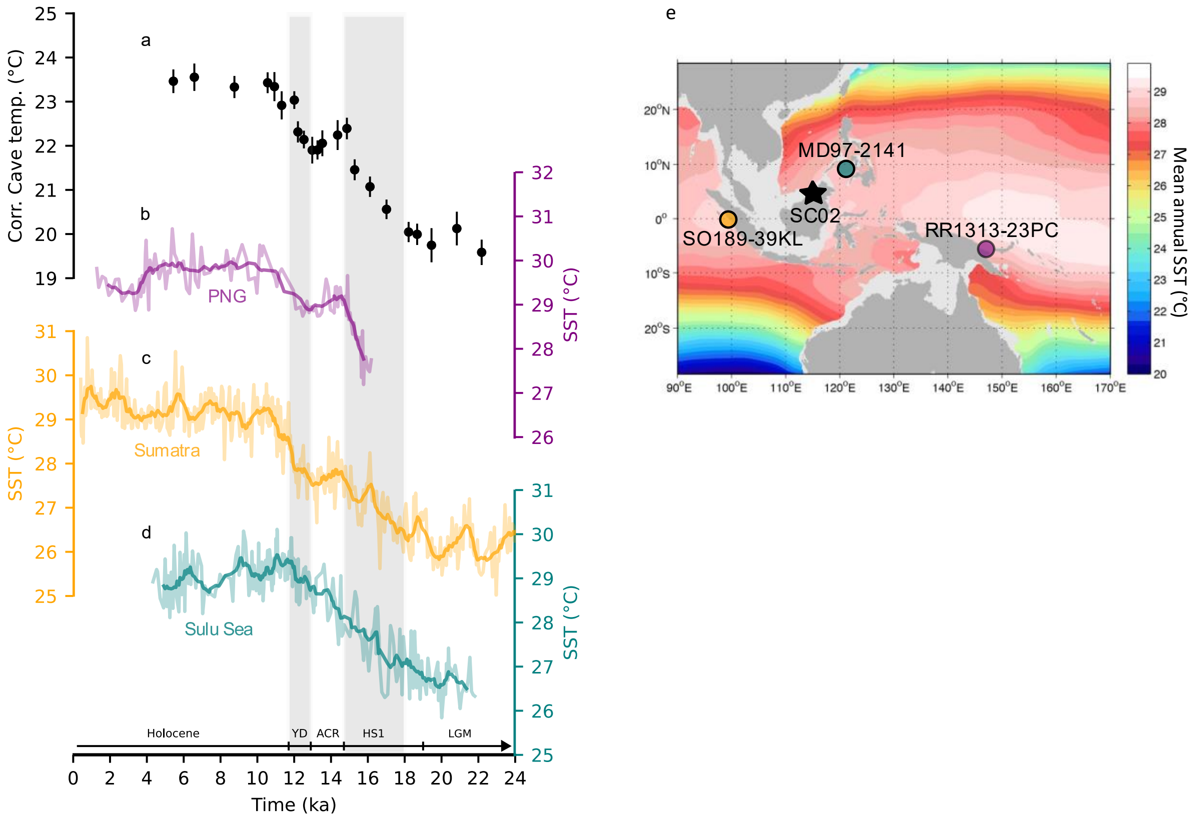

Supplementary Figure 3. Comparison of Borneo land temperature and regional SST records. a) Cave temperature record from this study corrected for changes of cave altitude relative to sea level. Error bars are 2 SEM. b) Mg/Ca-derived SST record from Papua New Guinea (RR1313-23PC)[4] with a 10-point moving average. c) Mg/Ca-derived SST record from western Sumatra (SO189-39KL)[5] with a 10-point moving average. d) Mg/Ca-derived SST record from the Sulu Sea (MD97-2141)[6] with a 10-point moving average. Grey vertical bars indicate pronounced NH cooling episodes. LGM: Last Glacial Maximum, HS1: Heinrich stadial 1, ACR: Antarctic Cold Reversal, YD: Younger Dryas. e) Map of present-day mean annual sea surface temperatures (SST), showing the location of SC02 and the SST records (modified from ref.[7]). Light grey area indicates land area exposed during the Last Glacial Maximum.

Supplementary Table 1. Overview of temperature data from Mulu Airport, Lang's Cave and Secret Chamber. Each location reports the time period and frequency of temperature recordings, as well as the temperature range and calculated average temperature. Temperatures at the entrance and inner parts of Lang's Cave were measured over multiple years using Onset HOBO U23-001 ProV2 loggers. Temperatures in Secret Chamber were measured using Van Essen CTD-Divers.

	Period	Recording interval	Range (°C)	Average temperature (°C)	Comments
Mulu Airport	03.07.2006-04.12.2012	daily	21.5-27.6	24.3	
Lang's Cave entrance	15.03.2005-30.12.2005	30 min	22.9-26.4	24.4	
Lang's Cave entrance	03.08.2006-02.04.2007	16 min	22.9-26.1	24.5	
Lang's Cave entrance	18.10.2012-28.10.2012	15 min	24.1-28.1	24.6	
Inner Lang's Cave	22.8.2008-12.3.2010	hourly	24.0-24.2	24.1	
Inner Lang's Cave	5.4.2018-23.9.2020	hourly	24.1-24.3	24.1	Corrected for +0.2 °C offset in cross-calibration
Secret Chamber, main	03.04.2018-02.04.2019	hourly	23.9-24.1	24.0	submerged CTD Diver
Secret Chamber, balcony	03.04.2018-02.04.2019	hourly	23.9-24.1	24.0	submerged CTD Diver

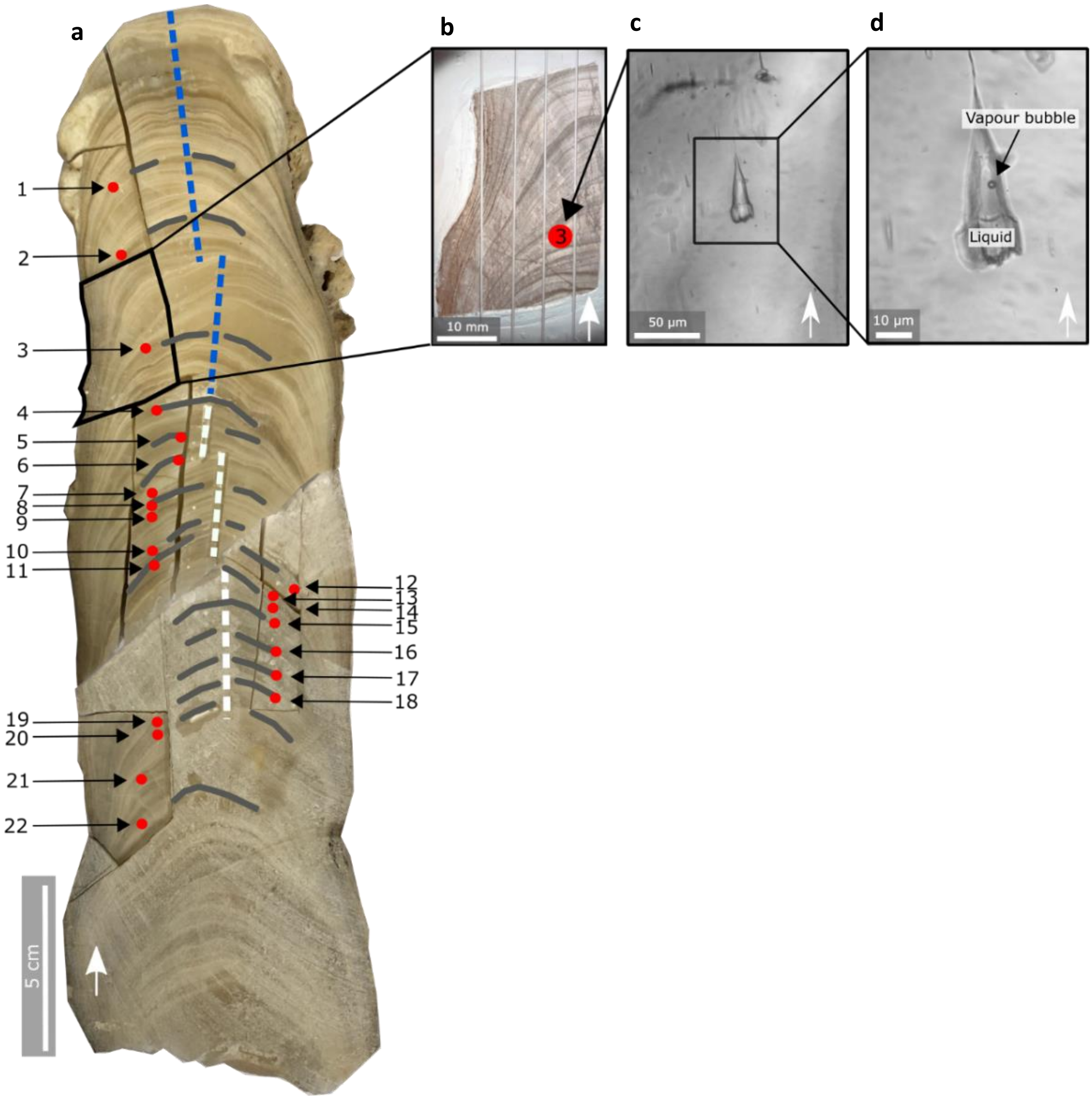

Supplementary Figure 4. Overview of stalagmite sample SC02. White arrows indicate growth direction. a) Red dots indicate the location of the samples analysed with fluid inclusion microthermometry. The stippled lines indicate the transects drilled for stable isotope measurements along the main growth axis. White: ref[8], blue: this study. Dark grey lines following the growth layers depict the location of samples milled out for U-Th dating[8]. Most of the fluid inclusion microthermometry measurements were performed on the same layers that were sampled for U-Th dating. To this end, blocks were cut from the side of the stalagmite slab and layers were optically traced sideways. b) Unpolished 300 µm thick stalagmite section for fluid inclusion microthermometry glued on a glass slide and cut into vertical stripes of 5 mm width. The red circle indicates the location of temperature point nr. 3. c) Image of a one-phase fluid inclusion used to measure liquid-vapour homogenisation temperature. d) Enlarged image of the fluid inclusion from c) in a two-phase state

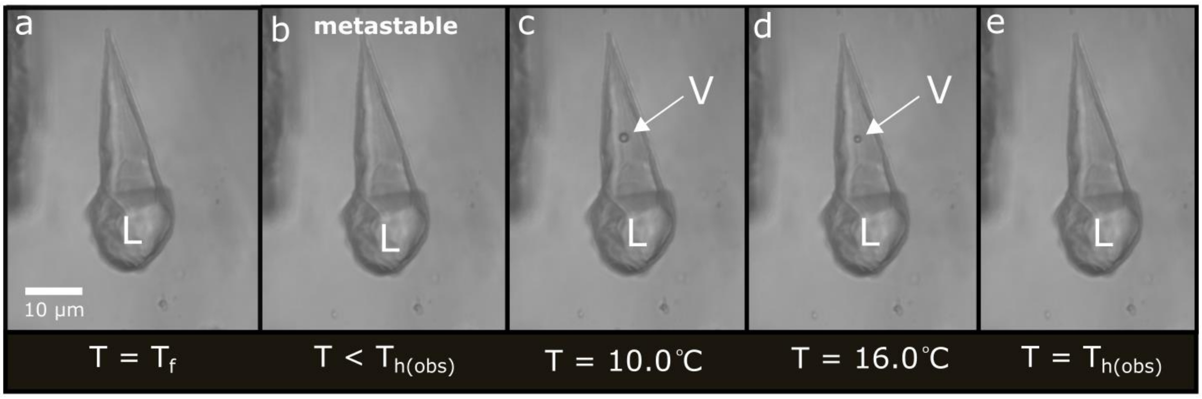

Supplementary Figure 5. Sequence of fluid inclusion images illustrating the different phase states during microthermometric measurements. L = liquid phase, V = vapour phase. a) Stable liquid state at the formation temperature T_f of the inclusion. b) Metastable liquid state at $T < T_{h(obs)}$. c) Stable liquid-vapour equilibrium state at 10.0 °C after laser-induced vapour bubble nucleation. d) Decrease of the vapour bubble size upon heating to 16.0 °C. e) Collapse of the vapour bubble and homogenisation to the liquid phase at $T_{h(obs)}$ of 18.3 °C. The calculated $T_{h\infty}$ is 19.8 °C and the volume of the inclusion is about 2200 µm^3.

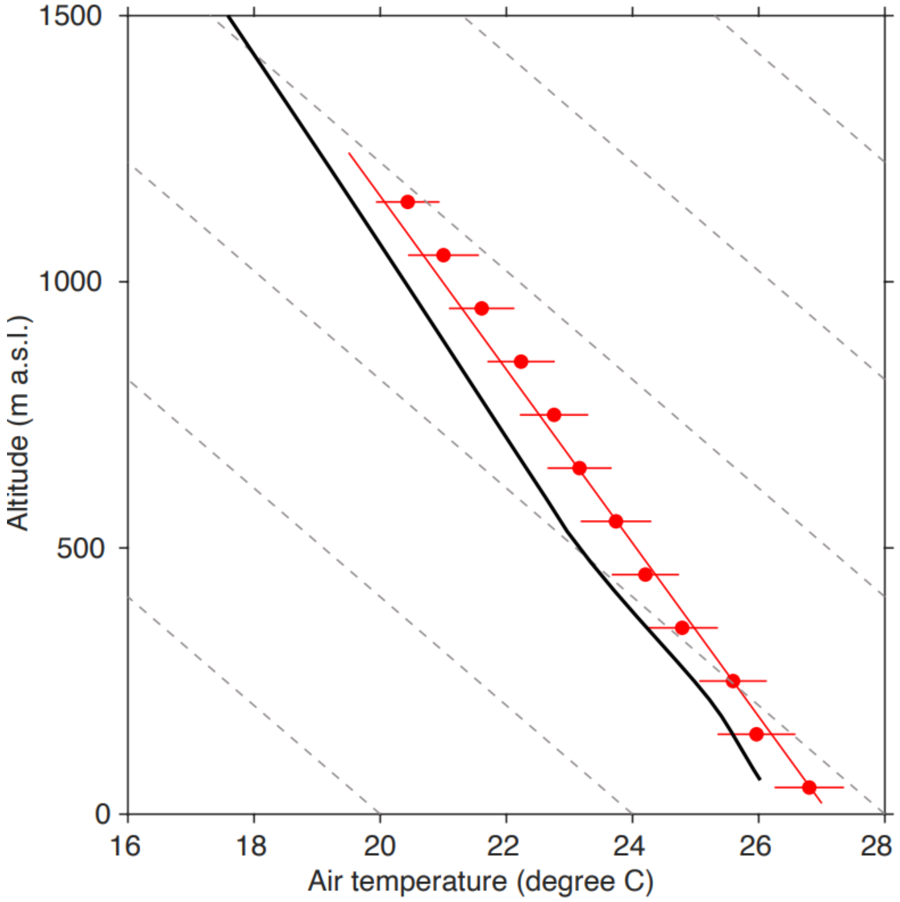

Supplementary Figure 6. Thermodynamic diagram of lapse rate estimates in the Mulu cave area. Red dots show binned means of 2 m (above ground) temperature from ERA5 data[9] within the area 114 – 118° E, 2.0 – 4.5° N for the period 1980 to 2019. Linear regression (red line) yields a lapse rate of Γ = 0.61 ± 0.002 °C/100 m (95% confidence bounds). Solid black line shows a free atmosphere mean air temperature profile for a grid point in the nearby low lands (114.15° E, 4.00° N) based on monthly mean fields in the same time period. Dashed grey lines indicate the dry adiabatic lapse rate (Γ = 0.98 °C/100 m).

References

1. Shakun, J. D. *et al.* Global warming preceded by increasing carbon dioxide concentrations during the last deglaciation. *Nature* **484**, 49, doi:10.1038/nature10915 (2012).
2. Osman, M. B. *et al.* Globally resolved surface temperatures since the Last Glacial Maximum. *Nature* **599**, 239-244, doi:10.1038/s41586-021-03984-4 (2021).
3. Bereiter, B., Shackleton, S., Baggenstos, D., Kawamura, K. & Severinghaus, J. Mean global ocean temperatures during the last glacial transition. *Nature* **553**, 39-44 (2018).
4. Moffa-Sanchez, P., Rosenthal, Y., Babila, T. L., Mohtadi, M. & Zhang, X. Temperature evolution of the Indo-Pacific Warm Pool over the Holocene and the last deglaciation. *Paleoceanography and Paleoclimatology* **34**, 1107-1123 (2019).
5. Mohtadi, M. *et al.* North Atlantic forcing of tropical Indian Ocean climate. *Nature* **509**, 76-80 (2014).
6. Rosenthal, Y., Oppo, D. W. & Linsley, B. K. The amplitude and phasing of climate change during the last deglaciation in the Sulu Sea, western equatorial Pacific. *Geophysical Research Letters* **30**, doi:10.1029/2002GL016612 (2003).
7. Meckler, A. N. *et al.* Glacial–interglacial temperature change in the tropical West Pacific: A comparison of stalagmite-based paleo-thermometers. *Quaternary Science Reviews* **127**, 90-116, doi:10.1016/j.quascirev.2015.06.015 (2015).
8. Buckingham, F. *et al.* Termination 1 Millennial-Scale Rainfall Events Over the Sunda Shelf. *Geophysical Research Letters* **49**, e2021GL096937 (2022).
9. Hersbach, H. *et al.* The ERA5 global reanalysis. *Quarterly Journal of the Royal Meteorological Society* **146**, 1999-2049 (2020).
